# Cationic hydrous thorium dioxide colloids – a useful tool for staining negatively charged surface matrices of bacteria for use in energy-filtered transmission electron microscopy

**DOI:** 10.1186/1471-2180-6-59

**Published:** 2006-06-27

**Authors:** Heinrich Lünsdorf, Ingeborg Kristen, Elke Barth

**Affiliations:** 1 GBF-National Institute of Biotechnology, Department of Environmental Microbiology, Mascheroder Weg 1, D-38124 Braunschweig, Germany

## Abstract

**Background:**

Synthesis of cationic hydrous thorium dioxide colloids (ca. 1.0 to 1.7 nm) has been originally described by Müller [22] and Groot [11] and these have been used by Groot to stain acidic glucosaminoglycans for ultrastructure research of different tissues by conventional transmission electron microscopy.

**Results:**

Synthesis of colloidal thorium dioxide has been modified and its use as a suitable stain of acidic mucopolysaccharides and other anionic biopolymers from bacteria, either as whole mount preparations or as preembedment labels, is described. The differences in stain behavior relative to commonly used rutheniumred-lysine and Alcian Blue™ electron dense acidic stains has been investigated and its use is exemplified for *Pseudomonas aeruginosa *adjacent cell wall biopolymers. For the first time thorificated biopolymers, i.e. bacterial outer cell wall layers, have been analysed at the ultrastructural level with electron energy loss spectroscopy (EELS) and electron spectroscopic imaging (ESI), leading to excellent contrast and signal strength for these extracellular biopolymers.

**Conclusion:**

Application of cationic hydrous ThO_2 _colloids for tracing acidic groups of the bacterial surface and/or EPS has been shown to be rather effective by transmission electron microscopy. Because of its high electron density and its good diffusibility it stains and outlines electro-negative charges within these biopolymers. In combination with ESI, based on integrated energy-filtered electron microscopy (EFTEM) Th-densities and thus negative charge densities can be discriminated from other elemental densities, especially in environmental samples, such as biofilms.

## Background

Capsular and slime matrices of pathogenic bacteria play an important role in host defense and the immune response. In particular the significance of neutral and/or acidic EPS in biofilms, either 'in vitro' or 'ex vitro', is known [31,3]. In this context it is of importance to obtain ultrastructural information on the amount and macromolecular architecture of the bacterial capsule and slime layers to allow morphological correlation with the physiological and pathogenic states in either 'in vitro' or 'in situ' situations. Identification of negatively charged matrices of bacteria at high resolution is possible through the use of transmission electron microscopy.

The use of colloidal thorium dioxide, i.e. Thorotrast™, as a high density stain for transmission electron microscopy began in the 1950s when colloids of 7 nm [10] were mainly used to trace the fate of Thorotrast™ in the tissues of mice and rats. Thorotrast™ was used in the late 1920s and early 1930s as a contrasting agent in vascular radiography in gastroenterology, bronchography and pyelography [32,2]. The uptake of thorium in the reticulo-endothelial system of spleen lead to a final deposition mainly in Kupffer cells of the liver [12,33]. Later colloidal thorium dioxide was used as an efficient high contrast agent that was rather specific for acidic glycoaminoglycans of the reticulo-endothelial system and cartilage [25,6,29]. In 1981 Groot [11] performed comparative studies of positively charged thorium dioxide colloids versus rutheniumn red and colloidal iron as contrast agents for glycoaminoglycans, i.e. hyaluronanes.

The usage of thorium dioxide as a stain of acidic mucopolysaccharides of bacteria and microorganisms, however, was only seldomly applied, either as pre-embedding or post-embedding label [35,34]. Since the advent of EFTEM as a potent tool for elemental microanalysis and element mapping of complex cell surface matrices at ultrastructural resolution, cationic thorium dioxide colloids seemed to be promising tracers for negatively charged groups or ligands because of their small size and good electron density.

The high binding strength and nearly quantitative binding of thorium(IV) to acidic biopolymers, especially those of the so-called particulate organic carbon (POC) in marine waters, has been known for a long time. Thus Th234 with its short half-life of 24 days, derived from the natural decay of uranium, has been used for global carbon flux measurements in the open oceans, based on the Th234/Th232 isotope ratio (Th232 half-life: 1.39 × 10^10 ^years)[28]. Actual studies revealed that Th(IV) binds not to only carboxylic groups, but also to phosphate and sulfate groups, and to EPS from different species of marine phytoplankton and bacteria (Alvarado-Quiroz et al.).

In the present paper we describe the preparation of cationic colloidal hydrous thorium dioxide, based on modified methods from Müller [22] and Groot [11]. Throughout the following text the term 'thorium dioxide' is used as a synonym of 'cationic hydrous thorium dioxide colloid'. Here, we give some applications on the staining of acidic cell wall exopolymers of *E.coli *and *Pseudomonas aeruginosa *by thorium dioxide and compare these with those from staining with ruthenium red or AlcianBlue™. Further high resolution thorium using EFTEM is presented. These experiments show thorium dioxide is a favorable alternative in contrasting acidic biomatrices relative to ruthenium red or AlcianBlue™.

## Results and discussion

**Figure 1:**
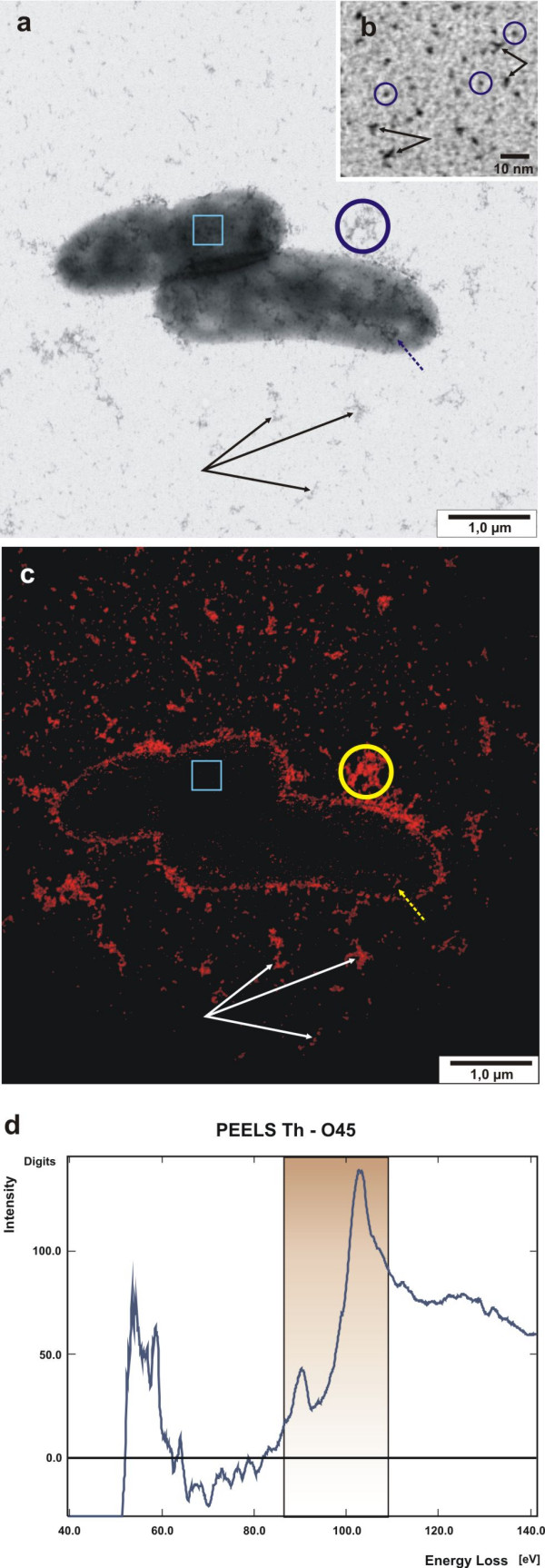
**Application of cationic hydrous thorium dioxide colloids for tracing acidic groups of bacterial slime matrices of whole mount *E. coli *cells**. (a) Bright-field image of mid-log *E. coli *cells which have been labelled with cationic 1.5 nm thorium dioxide colloids. Distinct irregular electron dense clusters of slime polymers are found associated with the cell surface (dashed arrow) or the cell periphery. Occasionally, slime is present as individual clusters in the background (triple arrows). Squared box indicates an area of high mass thickness, revealing low thorium intensities. (b) Detailled view of homogenous monodispersed particles of thorium dioxide; individual particles of 1.0 to 1.5 nm in diameter are circled, double arrows indicate oligoparticulate clusters. (c) High resolution elemental distribution map of thorium, corresponding to the bright-field image (a). (d) PEELS from an individual slime cluster, circled in (a) and (c). The boxed area in the spectrum record indicates the characteristic energy range of Th-O_4,5_, corresponding to the slit-width setting.

Some useful modifications, detailed below, have been introduced for the preparation of thorium dioxide by Müller [22] and Groot [11], that have made the final concentration of thorium dioxide more reliable. We also found the amount of thorium nitrate needed during clearance titration, i.e. peptization, is significantly smaller than originally described [22,11]. Following this procedure the preparation of thorium dioxide finally leads to a slightly opalescent solution as stated by Groot with colloidal particles ranging in size from 1.0 to 1.7 nm, with a mean diameter of 1.5 nm. As is shown in figure 1b the colloids appear monodispersed with high contrast down to individual particles. However, clusters with three or more particles are present, which give some indication of the chemical state of these Th (IV)-colloids. Formation of thorium dioxide under comparable conditions to those presented here, i.e. precipitation of thorium dioxide by alkali, has been reported by Dzimitrovicz et al. [4]. They determined a mean crystallite diameter of 1.6 nm by powder X-ray diffraction from the dried precipitate, which is in good agreement with actual measurements in the present study. However, based on direct high resolution electron microscopic studies of the dried ThO_2 _precipitate Dzimitrovicz et al. [4] described dimensions of 'particles' from 3 to 8 nm within the agglomerates. Similarly, Rothe et al. [26] prepared ThO_2 _× H_2_O(am) by alkaline precipitation with carbonate-free NaOH from thorium nitrate solution. They found the dried powder was amorphous, as X-ray powder diffraction patterns did not reveal any peaks or broadened bands, which is contradictory to the measurements of Dzimitrowicz et al. [4]. Rothe et al. [26] assumed a 2.4 water per ThO_2 _by gravimetric measurements after drying the sample at 800°C.

In general, the alkaline precipitate is considered to be a hydroxide sol, with a formula of either Th(OH)_4_(am) [23,19] or ThO_2 _× H_2_O(am) [27,9,24]. The specificity of thorium dioxide as a cationic positively charged particle is a consequence of peptization, which is carried out primarily with acid thorium nitrate, leading to deflocculation and thus to cationisation of the colloid by acidification. It is assumed that this is achieved by addition of protons, as discussed for the peptization of TiO_2 _[16]. Müller [22] has shown that final peptization with acidic thorium nitrate lead to a stable positively charged hydroxide sol, which was not impaired by the action of neutral salts.

Whole mount labelling of prefixed *Escherichia coli *cells showed distinct binding to negatively charged ligands, either directly associated with the cell surface or with peripheral amorphous slime (figure 1a).

Beside the excellent contrast these nanoparticles are ideally suited for EELS and ESI for high resolution imaging on Th-element mapping of the colloids (figure 1c). ESI is conveniently performed at the Th O_4,5_-edge at 98 to 102 eV and background intensity is corrected according to the 'Three-Window-Power-Law' method [5]. As shown in figure 1 the element specific intensities of the thorium map correlate precisely with the dark spots in the elastic bright-field view (fig. 1a relative to 1c), and structural details down to the individual colloid particle can be observed. However, those thorium dioxide particles or clusters, which are centrally positioned on the cell bodies and thus underlaid by the high mass thickness of the cytoplasm, are not revealed as element specific intensities (fig. 1a, b: squared box). The encircled area in figure 1a, c, indicates the area used for PEELS measurement, shown in figure 1d. The boxed area (fig. 1d) denotes the thorium O_4,5_-edge, which is similar to EELS reference measurements of slightly oxidized thorium particles described by Ahn et al. [1] and additionally outlines the energy slit width setting used.

**Figure 2:**
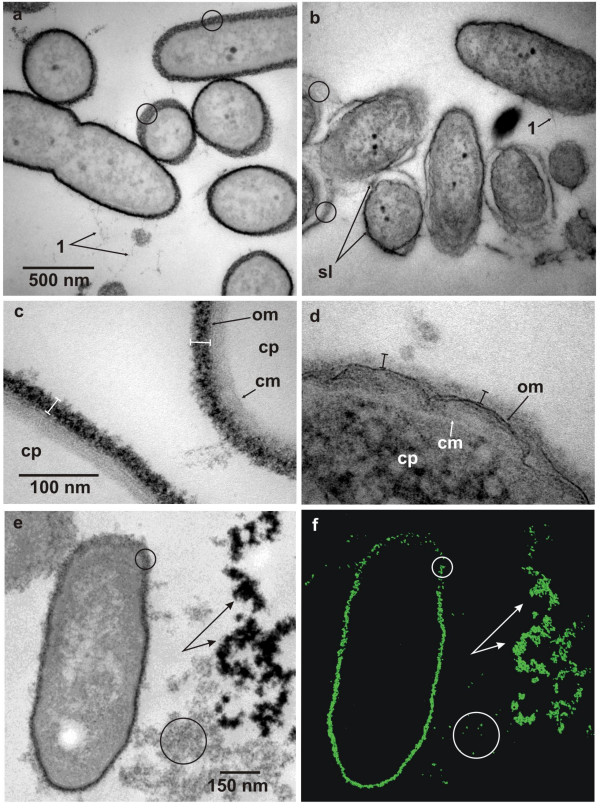
**Ultrathin sections of pre-embedding thorium dioxide label of cellular slime layers of *Pseudomonas aeruginosa***. (a) The outer surface of the cells is intensely stained with thorium dioxide colloids and from obliquely cut cells or cells cut non-equatorially this layer appears granular and non-fibrillar in substructure (circles). Fine fibrillar matter emanates from the cell surface (1) and shows a fine granular substructure. (b) Acidic mucopolysaccharide staining with AlcianBlue™. An electron dense slime layer (sl) envelopes the bacterial cells. This matrix irregularly contours the cell, either directly attaching the surface or running at varying distances. Obliquely cut layers show fibrillar rather than granular substructures (circles). (c) Detailled view of thorium dioxide label of the outer surface layer reveals a high density and granularity of slime layer (spacer bars), outer (om) and cytoplasmic membranes (cm) outline the periplasm, the cytoplasm (cp) is unstained and appears electron translucent. (d) Treatment of outer surface layer with RRL reveals a weakly greyish stained matrix (spacer bars). The outer (om) and cytoplasmic membranes (cm) are clearly visible as indicated, contouring the cytoplasm (cp). (e, f) Elastic bright field view (e) of a bacterial cell and adjacent EPS and the corresponding Th-elemental map (f) (section thickness: 35 nm). Because of the oblique orientation of the cell wall Th-densities in this area (small circles) are relatively low compared to the perpendicularly oriented parts. Large circles enclose EPS with low Th-densities and branched arrows indicate high Th-densities of EPS clusters with high negative intrinsic charges. Bars in (a, c, e) are valid for (b, d, f).

The practical potency of preembedding staining of acidic and/or negatively charged ligands of mucopolysaccharides is demonstrated in ultrathin sections (figure 2). Here *Pseudomonas aeruginosa *cells, growing in suspension, have been contrasted after aldehyde fixiation before embedment either with thorium dioxide (fig. 2a) or with AlcianBlue™ (fig. 2b). Apparently AlcianBlue™ in combination with uranylacetate 'en bloc'-staining lead to higher contrast intensities of the cytoplasm (fig. 2b), relative to the sole thorium dioxide-staining (fig. 2a). However, if one focuses on the cells' outer surface, acidic ligands are more intensely stained with thorium dioxide (fig. 2a), than with the AlcianBlue™ copper complex (fig. 2b). Additionally, because of the colloidal character of the thorium dioxide this stain shows distinct particulate granularity relative to the amorphous stain with AlcianBlue™. As should be noted further thorium dioxide is effectively excluded from the cytoplasm, which is strongly indicative for the cell wall's and cytoplasmic membrane's integrity during preembedding treatment. Further, thorificated cells appear similar in morphology relative to those which were conventionally stained. Additional ultrastructural differences of both stains are also obvious, when cell walls, which have been sectioned obliquely are presented as partial surface views (fig. 2a, b: circles). Differences are further exemplified by the granular decoration of slime fibrils, which extend from the cell surface (fig. 2a: 1), but which appear smoothly, when stained with AlcianBlue™ (fig. 2b: 1). Detachment of the surface layer (fig. 2a: sl) is obvious for the AlcianBlue™ stained cells.

The difference in staining of the outer wall-layers of *Pseuodmonas aeruginosa *with thorium dioxide compared to ruthenium red-lysine (RRL) is shown in figure 2c and 2d. Here views are given at higher magnification and reveal ultrastructural details of the outer cell surface, close to the macromolecular level. The high electron density of the polymer matrix outside of the outer membrane, which in the case of thorium dioxide staining is about 24 nm thick (fig. 2c), is indicative of the good permeation of the thorium colloids, which homogenously stain the matrix up to the outer membrane barrier. As is known from high-resolution ultrastructural analysis of freeze-substituted *Pseudomonas aeruginosa *biofilms, the intensely stained outer layer has been supposed to represent the B-band O-antigens of the lipopolysaccharide (LPS) layer, which is known to contain γ-D-mannuronic acid in substantial amounts [14,15].

In contrast this LPS-layer is only poorly stained by RRL, when method 2 of the Fassel-Edmiston protocol [7,8] was applied (see fig. 2d). Here, the 'outer layer' has a thickness of about 17 nm and thus has shrunken by about 30%, relative to the thorium dioxide stain (fig. 2c). Since ruthenium red has been described as a highly charged (+6) spherical particle of 1.1 nm in size [13] it is comparable in particle size and charge to thorium dioxide. The difference in contrast efficiency of thorium relative to ruthenium is obvious, even though RRL treated cells have been additionally osmicated and were conventionally poststained with uranylacetate and lead citrate. These differences in contrast-densities may arise as a consequence of the higher atomic mass (Th = 232; Ru = 101), more thorium atoms per particle volume or a higher binding strength of thorium to acidic groups relative to ruthenium atoms. It has been found that ruthenium red tends to be washed out during sample preparation, and because of this Luft [17,18] included this stain in all steps of his fixation protocol until the late dehydration steps.

Since no additional stain has been used during the embedding treatment with thorium dioxide all inner cellular biomatrices are visible from their own intrinsic contrast, based solely on mass thickness and elemental composition. In combination with EFTEM distinct ultrastructural features can be visualized either in the elastic bright-field view at 0 eV energy loss, termed 'zero-loss imaging' or 'high-contrast imaging' at 250 eV, revealing ultrastructures at high resolution with reversed intensities, similar to a dark-field view (data not shown). As shown here, zero-loss imaging by EFTEM renders the outer and cytoplasmic membrane as faint lines (fig. 2c, d).

The use of thorium dioxide is rather effective in tracing negatively charged surfaces or biomatrices with nearly a comparable diffusibility as non-colloidal, amorphous stain solutions. Therefore the corresponding charge densities found in images from thorium dioxide staining may correlate with grey level intensities of micrographs of zero-loss images. This is shown in different electron dense labels of LPS and EPS clusters in fig. 2e and the corresponding thorium distribution map in fig. 2f. Oblique orientation of labelled cell wall polymers (fig. 2e, f: small circles) reduces the specific stain densities within this particular sample volume and as a consequence the Th-intensity is reduced. Increase in net Th-intensity of perpendicularly oriented cell wall coatings is obvious. The differences in label densities of EPS clusters (fig. 2e, f: branched arrows, large circles) thus reflect the general aciditiy and/or low volume density of acidic groups within the EPS meshwork. However, it should be kept in mind that thorium dioxide labelling and staining only indicates general negatively charged ligands and will thus depend on the specificity or non-specificity of the underlying chemistry and on the pH of the milieu. Thus, at a low pH of 2.5 to 2.9 mainly acidic groups are traced, compared to a broad overall staining of acidic as well as neutral biomatrix components near neutral pH (data not shown).

## Conclusion

Synthesis of cationic hydrous colloidal thorium dioxide has been modified from former recipes and is described in detail. Its usage as an effective staining agent in transmission electron microscopy of acidic and negative groups of bacterial extracellular matrices has been reevaluated, both for preembedding treatment and 'whole mount' preparations. Its contrasting potency as a 1.5 nm particulate stain has been compaired with AlcianBlue™ and rutheniumred, which are commonly used to stain acidic mucopolysaccharides. The staining protocol is straight forward and as a 'one-component' stain there is minimum interference in contrast with other sample inherent elements, especially those in environmental probes. Charge densities appear directly modulated as corresponding grey values and are effectively traced as high resolution thorium-maps by energy-filtered transmission electron microscopy.

## Methods

### Synthesis of 1.5 nm cationic hydrous thorium dioxide colloids

(Modified from the procedures, described by Müller [22] and Groot [11]): 10 g Thorium nitrate hydrate (MM 480.06; Fluka, Switzerland) was dissolved in 50 ml reverse-phase osmophoresed water (20 % (w/v)), at a final pH of 2.4. To a volume of 20 ml thorium nitrate solution in a 250 ml round-bottomed flask, 0.4 ml aliquots of 25 % ammoniumhydroxide were added stepwise with continuous stirring. At pH = 3.0 the solution turned slightly turbid. Addition of a further 0.4 ml of NH_4_OH led to intense turbidity at pH = 4.0 and a further 0.4 ml of NH_4_OH quantitatively precipitated the thorium-hydroxide at a final pH = 11.0. The milky suspension was concentrated under slight vacuum over filter paper (grade 3 hw; Satorius, Göttingen, Germany) in a flat funnel and residual electrolytes were rigorously removed by washing with 100 to 150 ml reverse-phase osmophoresed water until no further ammoniac was evident. Thorium hydroxide was transfered to a 100 ml Erlenmeyer-flask by the aid of a spatulum. Residual precipitate was washed with 5 to 8 ml water and added to the bulk.

The slurry hydroxide was stirred and brought to boil under reflux. After 5 minutes 0.2 ml of thorium nitrate solution (20 % w/v) was added. This 'reflux-boiling' for 5 minutes was always performed, when 0.2 ml thorium nitrate aliquots had been added to the slurry. When a further 2 × 0.2 ml thorium nitrate had been added the solution clarified. Addition of a further 2 × 0.2 ml thorium nitrate led to slight re-turbification. A final addition of 0.2 ml thorium nitrate did not cause an increase in turbidity and this opalescent solution defined the final state of thorium dioxide. On the whole 1.2 to 1.6 ml of 20 % thorium nitrate were sufficient for thorium hydroxide peptization. The final volume was measured and the corresponding concentration of thorium dioxide was calculated relative to the original amount of Th(NO_3_)_4_. In general a 50 % (w/v) colloidal ThO_2 _solution was obtained with a pH of 2.0 to 2.5. On analysis by electron microscopy, thorium dioxide appeared monodispersed and showed a particle size, ranging from 1.0 to 1.7 nm in diameter. It could be stored in a refrigerator at 4°C for two years or even longer without loss of the colloidal state and charge properties.

### Whole mount staining of Escherichia coli with hydrous thorium dioxide colloids

Mid-logarithmically growing cells (4.0 ml) of *Escherichia coli*, strain EPI300-T1^R ^(Epicenter Biotechnologies, Madison, Wisconsin, USA) at an OD_590 nm _= 0.600 were sedimented at 7500 rpm (Biofuge Pico, Heraeus, Osterode, Germany) for 4 minutes at ambient temperature. Cells were resuspended in 1.0 ml of 1.8 % (v/v) formaldehyde – 10 mM Hepes, pH 7.1 and fixed for 15 minutes at ambient temperature, cells were again sedimented for 3 minutes and resuspended in 1.5 ml of 0.04 % (w/v) thorium dioxide, pH 3.0. Cells were subsequently incubated for 30 minutes at ambient temperature. Labelled cells were washed twice and finally resuspended in 200 μl 10 mM Hepes, pH 7.1.

### Preembedding label with hydrous thorium dioxide colloids

Mid-logarithmically growing cells of *Pseudomonas aeruginosa*, clinical strain PAO1 (NCCB 2425), were fixed with 2.5 %(v/v) glutardialdehyde – 20 mM Hepes, pH 7.0 for 2 hours or several days at ambient temperature. Cells were washed twice in 100 mM sodiumacetate, pH 3.0 for 15 minutes and then were labelled with 0.4 % (w/v) thorium dioxide in 100 mM sodium-acetate, pH 3.0 for 2 hours at ambient temperature or at 4°C over night. Labelled cells were washed once for 2 minutes at ambient temperature in 50 mM Hepes, pH 7.2 and then were immobilized in 2% aqueous agar. Immobilized bacteria were dehydrated in an acetone-series and were embedded in epoxy resin according to Spurr [30].

### Preembedding label with AlcianBlue™

*Pseudomonas aeruginosa *mid-logarithmically growing cells were prefixed with 2.5 % (v/v) glutardialdehyde in 10 mM Hepes, pH 7.2 after immobilization in aqueous 1% (w/v) agar, and were treated with AlcianBlue™ by aid of a microwave oven as described by Moorlag et al. [21].

### Preembedding label with ruthenium red-lysine

*Pseudomonas aeruginosa *mid-logarithmically growing cells were treated for staining and embedding as described by Fassel and Edmiston [7].

### Transmission electron microscopy

#### (a) Whole mount cell preparates

Thorium dioxide labelled cells were adsorbed for 2 minutes at carbon-Formvar (thickness: 60 nm to 90 nm) coated 300 mesh Cu-grids and were intensely blotted with filter paper (grade 3 hw; Satorius, Göttingen, Germany) and air-dried. Transmission electron microscopy and EELS analysis were performed with a Zeiss CEM902. Recording of thorium maps by ESI was performed according to the 'Three-Window-Power-Law' method (65 eV, 85 eV, 112 eV) with an energy slit-width of 24 eV and an objective aperture of 6 mrad (= 30 μm). PEELS were recorded in an energy range from 40.0 to 140.0 eV. Element maps and PEELS were recorded with a cooled 1024 × 1024 CCD camera (Proscan CCD HSS 512/1024; Proscan Electronic Systems, Scheuring, Germany).

#### (b) Ultrathin sections of embedded cells

Ultrathin sections (90 nm) were produced and analyzed by transmission electron microscopy as is described in detail by Lünsdorf et al. [19]. Thorium mapping was performed with an instrument setting as is described above, analyzing ultrathin sections of 35 nm thickness. Electron micrographs were registered with a cooled 1024 × 1024 CCD camera (Proscan CCD HSS 512/1024; Proscan Electronic Systems, Scheuring, Germany).

## Abbreviations

EPS: extracellular polymeric substances; EFTEM: Energy-filtered transmission electron microscopy; EELS: electron energy loss spectroscopy; PEELS: parallel EELS; ESI: electron spectroscopic imaging;

## Authors' contributions

IK and EB performed the synthesis of thorium dioxide and its application for transmission electron microscopy. HL designed the experiments, performed EFTEM-analysis and wrote the manuscript.
